# Supplementary material for: Personal Passive Air Samplers for Chlorinated Gases Generated from the Use of Consumer Products
Source: Int J Environ Res Public Health. 2021 Aug 25;18(17):8940. doi: 10.3390/ijerph18178940 (PMC8430877; doi:10.3390/ijerph18178940)
Supplement: Supplementary file 1 [file ijerph-18-08940-s001.zip › ijerph-1334315-Supplementary.pdf]

Supplementary material

Personal passive air samplers for chlorinated gases generated from the use of consumer products

*YEONJEONG HA, YERIM KOO, and JUNG-HWAN KWON**

Division of Environmental Science and Ecological Engineering, Korea University, 145 Anam-ro, Seongbuk-gu, Seoul 02841, Republic of Korea

***Corresponding Author**

Phone: +82 2 3290 3041

E-mail: junghwankwon@korea.ac.kr

ORCID: 0000-0002-6341-7562

Table S1. Raw response of PTR/SRI-MS, observed chlorine isotope ratios, and chlorine concentration at each time point shown in Figure 1

Time (min)	0		5		15		30		60		120	
m/z	[a]PTR-MS response	[b]ratio	PTR-MS response	ratio	PTR-MS response	ratio	PTR-MS response	ratio	PTR-MS response	ratio	PTR-MS response	ratio
70	33.6	7.5	30.4	7.9	37.5	7.8	44.5	12.3	53.3	12.3	67.8	9.2
72	17.8	4.0	21.0	5.5	24.5	5.1	28.5	7.9	31.8	7.3	42.1	5.7
74	4.5	1.0	3.8	1.0	4.8	1.0	3.6	1.0	4.3	1.0	7.4	1.0
[c]Σ	55.9	-	55.3	-	66.8	-	76.7	-	89.5	-	117.2	-
[d]C_{Cl2} (ppbv)	552.46		546.06		660.27		758.14		884.57		1158.42	

[a] Raw response of PTR/SRI-MS measurement at each passive sampler deployment time. The raw responses were calculated by subtracting responses of outside chamber (background level) from inside chamber that contains liquid bleach at the bottom.

[b] Observed chlorine isotope ratios. Chlorine isotope $^{35}Cl^{35}Cl^-$, $^{37}Cl^{35}Cl^-$ or $^{35}Cl^{37}Cl^-$, and $^{37}Cl^{37}Cl^-$ have m/z values of 70, 72, and 74, respectively. The expected chlorine isotope ratio is m/z 70: 72: 74 = 9:6:1.

[c] Summation of PTR/SRI-MS raw response occurred at m/z=70,72, and 74.

[d] The chlorine concentration was calculated by the calibration curve of PTR/SRI-MS of chlorine gas described in our previous study[1] (PTR/SRI-MS response = 0.1012 × C_{Cl2} (chlorine concentration), r^2=0.9942)

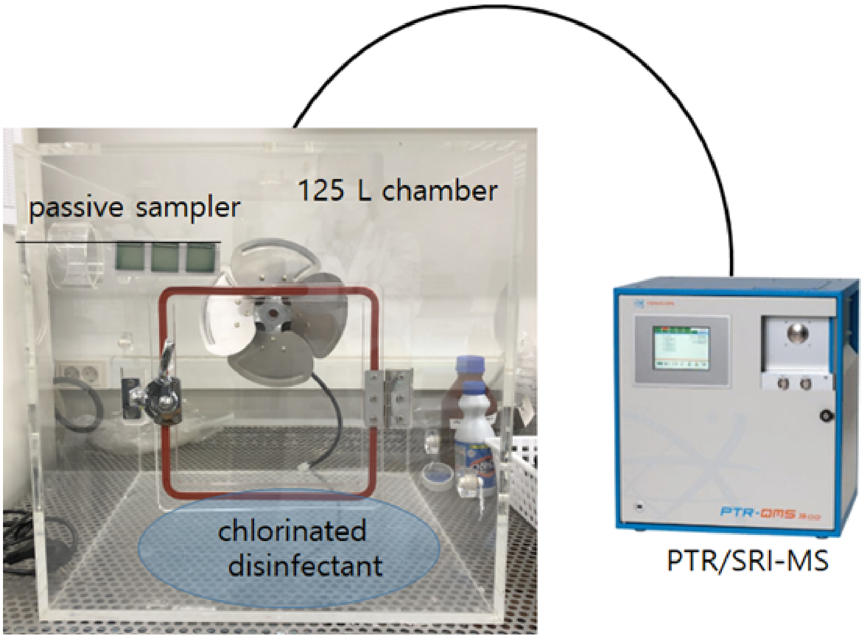

Figure S1. Scheme of the chamber design used in this study. Passive sampler patches were hung on the top of the 125 L acryl chamber and chlorinated disinfectants were spread on the bottom of the chamber. Proton transfer reaction/selective reagent ionization-mass spectrometer (PTR/SRI-MS) was connected on the top of the chamber to measure chlorine concentration.

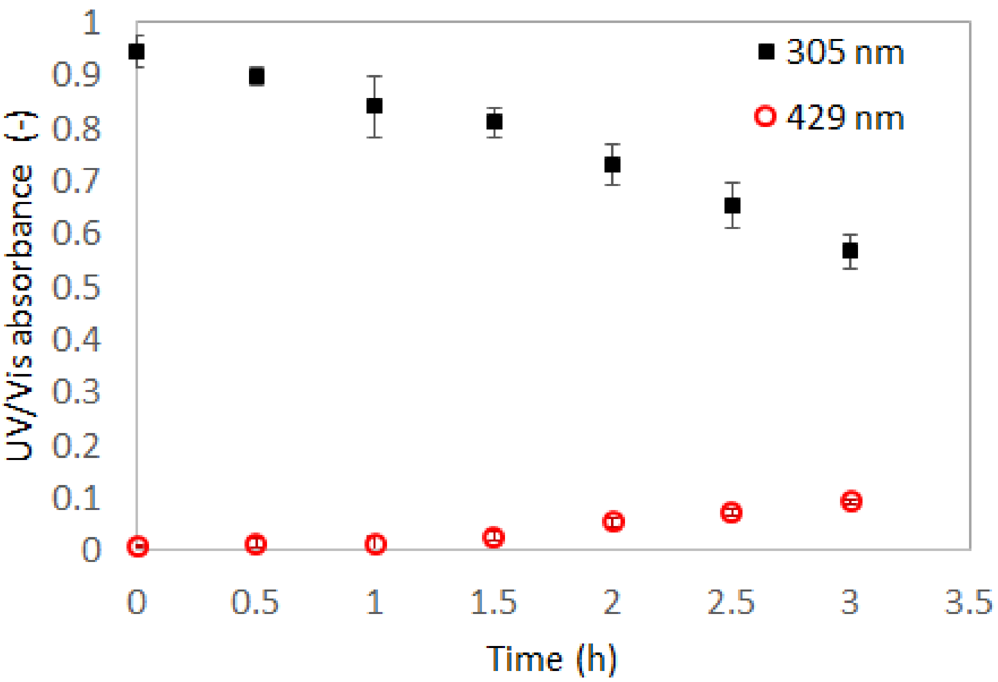

Figure S2. UV/Vis absorbance of passive sampler extraction solvent (isopropyl alcohol) at 305 nm (o-dianidisine) and 429 nm (oxidized form of o-dianisidine synthesized by oxidation reaction of o-dianisidine with chlorine) after passive sampler deployment in 125 L chamber with spreading 50.8 g of a gel type of chlorinated disinfectant.

References

1. Ha, Y., Kim, Y., Song, E., Yoo, H. J. & Kwon, J. H. Development of a personal passive air sampler for estimating exposure to effective chlorine while using chlorine-based disinfectants. *Indoor air* **31**, 557-565 (2021).
